# Supplementary material for: Patterns of Intron Gain and Loss in Fungi
Source: PLoS Biol. 2004 Nov 30;2(12):e422. doi: 10.1371/journal.pbio.0020422 (PMC532390; doi:10.1371/journal.pbio.0020422)
Supplement: Table S1 — Also available at http://genes.mit.edu/NielsenEtAl/. (4.3 MB ZIP). [file pbio.0020422.st001.zip › NielsenEtAl/html/960.html]

AN1442.1.NCU02681.1.MG04412.1.FG01215.1


```
 CLUSTAL W (1.82) Multiple Sequence Alignments - Introns Inserted


Sequence 1: NCU02681.1	221 aa
Sequence 2: MG04412.1	262 aa
Sequence 3: FG01215.1	215 aa
Sequence 4: AN1442.1	272 aa
Alignment Length: 287 aa
Number Identitical Residues: 93 aa
Alignment Score (without introns) 5033


MG04412.1 	MFGIDWMSLVLPFSYILVLGGTFMTFSSVYRKRKAT1QSANLAPWFPPHLQRNIYLSLLH
NCU02681.1	MFSVDWRGLVLPFAYLIVLTGTFMTFSTIYRKRKAQ1QSANLAPWFGPHLQRNVYLSLLH
FG01215.1 	MFNLDWN-DLLPLAYVAVLGGSLMAFSTIYRKRQAA1ESANLAPWFGPHLQRNVYLSLLH
AN1442.1  	--MVDWVTLIVPFAYLSVLIGSLATFSSLYRKRKAR1KATSLEPWFPSHLQRDIYFSLLH
          	   :**   ::*::*: ** *:: :**::****:*  :::.* *** .****::*:****

MG04412.1 	LDP-EDGSEKAPKVPESVLRTALLRRAVEDIHRIIQIRTAKAACSSLLARGSVGDDLWQR
NCU02681.1	MEP-ENGSEKAPKVPESVLRAALLRRAVEDIHRIIQIRAAKAACSSLLQRGSIGDDLWQR
FG01215.1 	LD---DGSEKSPKVPESVLRAALLRRAVEDIRRLIQVKSAKQACGSLLAKGSVGDDLWQR
AN1442.1  	LDPPASATEKKVAVPESVLKAALLRRAQEDIRRVMTLRSQKQALSVLLQRGSVGDDLWQR
          	::.. ..:**   ******::****** ***:*:: ::: * * . ** :**:*******

MG04412.1 	FSRAEKEMEEELRDVVME0ANALAPNWGQTIFQSANEIAANTMLRKRLDDITNQTEAEKE
NCU02681.1	FQRAEKEMEEELRDVVME0ANALAPNWGQSIFQTANEIAANTVLRERLDEIQAQATQEKE
FG01215.1 	FQRAEKEMEEELRDVVTE0ANALAPNWGQSIFQSAHEMTANAAMRSSIEEILAQADSEKQ
AN1442.1  	FLRAEKEMEDEVRDVVTE0ANAYAPNWGQVIFQSANEMVNNEIVRERLDKHQKKLDEEKA
          	* *******:*:**** * *** ****** ***:*:*:. *  :*. ::.   :   ** 

MG04412.1 	WWAKRQGAAGIEQPAASSPTTTTSESLPATSKETPGSSEDGILVEDDVPESS-------T
NCU02681.1	WWEKRR-------------ATVRSEFMKELEGDEAGTG----------------------
FG01215.1 	WWEKRR-------------GQIQTEFMKELDSEQSSSAK---------------------
AN1442.1  	WWEKKK-------------ASIQEGFMKELDAEKPGTVTTAAAATAAPEVAEGTQVAVGS
          	** *::                    :   . : ..:   .         :..:. : .:

MG04412.1 	PAPAAPAPVQTPSAPPSEP-----------------GTPSSKKKKKGKK
NCU02681.1	------TPVDTSSVTSS---------------------PSNKKGKN---
FG01215.1 	------ATSEEDAVLVED--------------------PSN--------
AN1442.1  	KASASAAAPPAPTVTESDDDAVLVEADDQAGSAGQKSPGGGKKKKKGKK
          	 :.::.:.    :.  .. .:   .:...:.::..... .... .....
```
